# Video-telehealth to support clinical assessment and management of acutely unwell older people in Residential Aged Care: a pre-post intervention study

**DOI:** 10.1186/s12877-021-02703-y

**Published:** 2022-01-10

**Authors:** Carolyn Hullick, Jane Conway, Alix Hall, Wendy Murdoch, Janean Cole, Jacqueline Hewitt, Christopher Oldmeadow, John Attia

**Affiliations:** 1 School of Medicine and Public Health, University of Newcastle, Callaghan, Australia; 2 Belmont Hospital, Hunter New England Local Health District, Belmont, Australia; 3 Hunter Medical Research Institute, New Lambton Heights, Australia; 4 John Hunter Hospital, Hunter New England Health, Locked Bag 1, HRMC, New Lambton Heights, NSW 2310 Australia; 5 Anglican Care, Booragul, Australia; 6 Hunter New England Central Coast Primary Health Network, Newcastle West, Australia

**Keywords:** Telemedicine, Long-term care, Geriatric emergency medicine, Aged, Avoidable hospitalization, Nursing, Telehealth, Implementation science, Aged 80 and over, Transitional care

## Abstract

**Background:**

Older people living in Residential Aged Care (RAC) are at high risk of clinical deterioration. Telehealth has the potential to provide timely, patient-centred care where transfer to hospital can be a burden and avoided. The extent to which video telehealth is superior to other forms of telecommunication and its impact on management of acutely unwell residents in aged care facilities has not been explored previously.

**Methods:**

In this study, video-telehealth consultation was added to an existing program, the Aged Care Emergency (ACE) program, aiming at further reducing Emergency Department (ED) visits and hospital admissions. This controlled pre-post study introduced video-telehealth consultation as an additional component to the ACE program for acutely unwell residents in RACs. Usual practice is for RACs and ACE to liaise via telephone. During the study, when the intervention RACs called the ED advanced practice nurse, video-telehealth supported clinical assessment and management. Five intervention RACs were compared with eight control RACs, all of whom refer to one community hospital in regional New South Wales, Australia. Fourteen months pre-video-telehealth was compared with 14 months post-video-telehealth using generalized linear mixed models for hospital admissions after an ED visit and ED visits. One thousand two hundred seventy-one ED visits occurred over the 28-month study period with 739 subsequent hospital admissions.

**Results:**

There were no significant differences in hospital admission or ED visits after the introduction of video-telehealth; adjusted incident rate ratios (IRR) were 0.98 (confidence interval (CI) 0.55 to 1.77) and 0.89 (95% CI 0.53 to 1.47) respectively.

**Conclusions:**

Video-telehealth did not show any incremental benefit when added to a structured hospital avoidance program with nursing telephone support.

**Trial registration:**

The larger Aged Care Emergency evaluation is registered with ANZ Clinical Trials Registry, ACTRN12616000588493.

## Background

Telehealth is the use of information technology, specifically the addition of video-conferencing, for clinical care when distance and/or time separate the patient and the healthcare provider [1]. It has the potential to provide frail, older adults, particularly those living in Residential Aged Care (RAC), with timely, patient-centered care where transfer to hospital can itself be a burden [2] and potentially avoided. Telehealth is proposed as an important part of contemporary models of care for geriatric emergency medicine [3] and has the potential to improve access to care [4].

In 2018, 7.6% of Australians over the age of 65 lived in residential care [5]. RAC residents have higher risks of hospitalization, and Emergency Department (ED) visits [6–10] than older people who are living in the community [11]. Older people in RACs are living the last years of their life. They are at high risk of acute clinical deterioration, often complicated by frailty and multi-morbidity [5]. Hospitalization risk [11] includes delirium, pressure injury, falls, sarcopenia [12] and medication errors [11]. Programs to better support their healthcare needs in the RAC and avoid transfer to the ED, mitigate this risk [13]. It is also important to recognise that when avoiding hospitalization, residents of RACs should still receive the acute care they require, in line with their goals of care.

Transitional care is a set of actions that promotes continuity, avoids poor outcomes and promotes timely and safe transfer of patients at critical exchange points, from one type or level of healthcare to another [14]. The Aged Care Emergency (ACE) program is a collaboration between RACs, the Local Health District, and primary health organizations aiming to avoid hospitalization and support transitional care to manage acutely unwell residents [13]. It includes 24-h nurse-led telephone support with evidence-based algorithms for common symptoms and problems. When transfer is required, the reason for the transfer and the resident’s goals of care are clarified. The ACE program has engaged 81 RACs that primarily transfer residents to nine EDs across a large regional Local Health District (LHD) of Northern and Western New South Wales (NSW), Australia, including regional and rural communities. Evaluation of the implementation of the program across the region has shown a 20% reduction in ED visits and hospitalizations [13]. A local organization with several RACs approached their ACE partnering hospital to trial telehealth as an additional component to the ACE program.

The objective of this study was to determine if adding video-telehealth consultation to the established ACE program further reduced ED visits and hospital admissions.

## Methods

### Study design

This exploratory study was a controlled pre-post study conducted after the completion of a larger stepped wedge non-randomized trial, which assessed the effect of the Aged Care Emergency (ACE) program [13]. The pre-phase represented 14 months of hospital data collected prior to the delivery of the video-telehealth strategy (i.e. ACE program alone), while the post-phase represents the 14 months of hospital data collected from the time of implementation of the video-telehealth strategy (i.e. ACE program plus video-telehealth).

### Study setting and participants

Video-telehealth was introduced in Lake Macquarie, NSW, Australia using the 13 RACs referring patients to the same community Hospital ED (Belmont, NSW) as part of the ACE program. The five intervention RACs all belonged to one not-for-profit aged care organization. The remaining eight RACs acted as control and did not use video-telehealth as part of the program. Table 1 has further details regarding RAC number of beds, access to registered nurses (RNs), dementia-specific beds and respite beds.

**Table 1:** Residential Aged Care: characteristics of facilities

		Telehealth		
Variable		No (***n*** = 8)	Yes (***n*** = 5)	Total (***N*** = 13)
Dementia care	Yes	3 (38%)	3 (60%)	6 (46%)
Respite	Yes	5 (63%)	3 (60%)	8 (62%)
24/7 RN	Yes	7 (88%)	4 (80%)	11 (85%)
Number of beds	median (Q1, Q3)	71 (54, 99)	60 (42, 63)	60 (52, 91)

The ACE hospital avoidance program had been implemented in both telehealth and control sites at least 9 months prior to data collected for this study (i.e. 23 months prior to commencement of the intervention).

### Intervention

The telehealth intervention was activated in two clinical scenarios:

#### Acute change in condition that may require transfer to hospital

For the five intervention RACs, when the RAC called the ED aged care RN associated with the ACE program, the video-telehealth pathway was only activated 7-days a week for calls between 8 am to 4 pm. This involved two-way, real-time interactive communication between the resident with support from RAC staff and ED [15]. The RAC staff member activated the call, managed the patient, managed the camera and equipment and undertook tasks to support the telehealth call. They also considered the recommendations from the ED RN regarding management including maintenance in the RAC or transfer to the ED. Out of hours, telephone support continued to be provided by the primary health organization as part of the ACE program, with no video enhancement [13].

#### Clinical handover from hospital to RAC

When a new or returning patient was being transferred to the RAC, a planned video-telehealth enhanced clinical handover occurred while the patient was in hospital with a booked appointment. A follow-up phone call occurred within the next week to provide clarification of clinical handover between the RAC RN and the hospital RN. Control sites continued to have telephone clinical handover.

#### Equipment, telehealth support and training

When the resident was unwell, a portable computer tablet device that could be held by the resident with nursing support was taken to the resident’s bedside. In the hospital, the ED aged care RNs and ward RNs used a computer on wheels with external speakers to enhance sound quality. Scopia software [16] was used as it is web-based, secure and downloaded rapidly if family or caregivers were included in the call. The LHD telehealth personnel were available by telephone when required; they recommended equipment and assisted with training both hospital and RAC staff. An advanced practice RN supported the program 1 day a week including trouble shooting, change management and training as well as monthly project meetings with stakeholders. The RN trained the RAC and hospital administrative and nursing staff. Stakeholders included a nurse educator and nurse practitioner from the RAC organization, the ACE program advanced practice RN, LHD telehealth support as well as ED and older persons’ ward representatives from the hospital.

### Data collection

Patient clinical and demographic characteristics, ED visits for all the hospitals across of the LHD, admission status, admission diagnosis, data relating to ED visits, and subsequent hospital admissions were collected on a monthly basis. Data 14 months before the intervention and 14 months after the intervention, from May 2015 to August 2017 were analyzed.

### Outcomes

The primary outcome was hospital admissions and secondary outcome was ED visits.

### Statistical analysis

Descriptive statistics were used to describe the study sample and main study outcomes. They are presented overall and separately by telehealth group for the pre- and post-telehealth phase in Table 2.

**Table 2:** Characteristics of Emergency Department visits by telehealth group pre and post-delivery of the telehealth strategy

Variable		Pre-intervention control (***n*** = 461)	Post-intervention control (***n*** = 435)	Pre-intervention telehealth (***n*** = 201)	Post -intervention telehealth (***n*** = 174)	Total (***N*** = 1271)
Sex	Female	297 (64%)	291 (67%)	133 (66%)	121 (70%)	842 (66%)
Arrived by ambulance	Yes	423 (92%)	403 (93%)	185 (92%)	166 (95%)	1177 (93%)
Triage category	Resuscitation	9 (2.0%)	4 (0.9%)	0 (0%)	3 (1.7%)	16 (1.3%)
	Emergency	62 (13%)	74 (17%)	26 (13%)	26 (15%)	188 (15%)
	Urgent	153 (33%)	141 (32%)	73 (36%)	56 (32%)	423 (33%)
	Semi-urgent	215 (47%)	202 (46%)	92 (46%)	84 (48%)	593 (47%)
	Non-urgent	22 (4.8%)	14 (3.2%)	10 (5.0%)	5 (2.9%)	51 (4.0%)
Disposition	Critical care ward	22 (4.8%)	22 (5.1%)	10 (5.0%)	12 (6.9%)	66 (5.2%)
	Discharged	190 (41%)	154 (35%)	75 (37%)	57 (33%)	476 (37%)
	General ward	232(50%)	229 (53%)	113 (56%)	99 (57%)	673 (53%)
	Transferred to other hospital	14 (3.0%)	20 (4.6%)	3 (1.5%)	6 (3.4%)	43 (3.4%)
Triage diagnosis	Carer Concern	28 (6.3%)	22 (5.4%)	6 (3.1%)	8 (4.8%)	64 (5.3%)
	Chest pain	17 (3.8%)	10 (2.5%)	10 (5.2%)	8 (4.8%)	45 (3.7%)
	Collapse/Syncope	11 (2.5%)	9 (2.2%)	6 (3.1%)	2 (1.2%)	28 (2.3%)
	Confusion	22 (5.0%)	26 (6.4%)	8 (4.1%)	6 (3.6%)	62 (5.1%)
	Fall, Unspecified	114 (26%)	115 (28%)	54 (28%)	53 (32%)	336 (28%)
	Fever	9 (2.0%)	20 (4.9%)	4 (2.1%)	6 (3.6%)	39 (3.2%)
	Injury	26 (5.9%)	18 (4.4%)	14 (7.3%)	14 (8.4%)	72 (6.0%)
	Other	151 (34%)	117 (29%)	64 (33%)	42 (25%)	374 (31%)
	Pain - Abdominal	12 (2.7%)	11 (2.7%)	7 (3.6%)	5 (3.0%)	35 (2.9%)
	Respiratory - Cough	10 (2.3%)	14 (3.5%)	4 (2.1%)	8 (4.8%)	36 (3.0%)
	Respiratory - Shortness of Breath	33 (7.4%)	33 (8.1%)	11 (5.7%)	12 (7.2%)	89 (7.4%)
	Urinary Problems	11 (2.5%)	10 (2.5%)	5 (2.6%)	3 (1.8%)	29 (2.4%)
Died in hospital	Yes	29 (6.3%)	53 (12%)	12 (6.0%)	13 (7.5%)	107(7.9%)
Age	median (Q1, Q3)	87 (83, 91)	87 (82, 91)	87 (83, 91)	89 (84, 92)	87 (83, 91)
Length of stay ED (mins)	median (Q1, Q3)	369 (246, 566)	337 (235, 508)	350 (239, 587)	371 (255, 518)	356 (239, 549)

Generalized linear mixed models (GLMM) assuming a negative binomial distribution and applying a log-link function were used to estimate differences in changes over time between control and telehealth RACs in the log value of the following outcomes: (1) number of inpatient admissions per month; (2) number of ED visits per month. Negative binomial models were used as the outcomes were all count variables that illustrated overdispersion. All models included a random intercept for RAC to account for clustering by RAC, and fixed effects for: receipt of the telehealth strategy (yes vs. no), phase of the study (pre-telehealth phase vs. post-telehealth phase) and receipt of the telehealth strategy by telehealth phase interaction term. A model adjusting for RAC characteristics was also conducted to control for potential confounding due to the non-randomized design of the trial. In the adjusted models the following RAC characteristics were included as fixed effects: dementia care, respite beds and presence of 24 h-a-day 7 days-a-week RNs (see Table 1). Furthermore, an offset of the log number of beds per RAC was also included in all models to account for variation in the number of beds between RACs.

The incidence rate ratios (IRR) (i.e. exponentiated parameter estimates) and 95% Confidence Intervals (CI) from the unadjusted and adjusted models are presented for the change in outcomes from pre to post-telehealth phase for both groups and the receipt of telehealth strategy by telehealth phase interaction. The *p*-value from the adjusted model is presented for the interaction term. A significant difference in the telehealth phase by receipt of telehealth strategy interaction effect at the 0.05 p-value was used to evaluate the effectiveness of the telehealth strategy.

### Sample size

Based on 160 admissions per year from 393 beds, we estimated a ~ 40% admission incidence density; this number of beds gave us 80% power at *p* = 0.05 to detect a 10% difference in admissions.

### Ethics

The study was reviewed and approved as low and negligible risk by the Hunter New England Human Research Ethics Committee. Individual patient consent was not required. LNR/14/HNE/242-14/06/18/5.10. Our research was performed in accordance with the Declaration of Helsinki and its relevant guidelines and regulations.

## Results

As indicated in Table 1, the five telehealth intervention and eight control RACs had similar characteristics except for dementia beds. There was a total of 733 hospital admissions across the 28-month period of the study for the 13 RACs; 226 from RACs that received intervention (115 in the pre-telehealth phase and 111 in the post-telehealth phase) and 507 from RACs that did not receive the telehealth strategy (249 in the pre-telehealth phase and 258 in the post-telehealth phase). One thousand two hundred seventy-one ED visits occurred, 375 from sites that received the telehealth intervention strategy (201 in the pre telehealth phase and 174 in the post telehealth phase); and 896 occurred in the control sites (461 in the pre-telehealth phase and 435 in the post telehealth phase). Table 2 details the demographic and clinical characteristics of the ED visits that occurred for sites taking part in the telehealth study by phase.

**Fig. 1:**
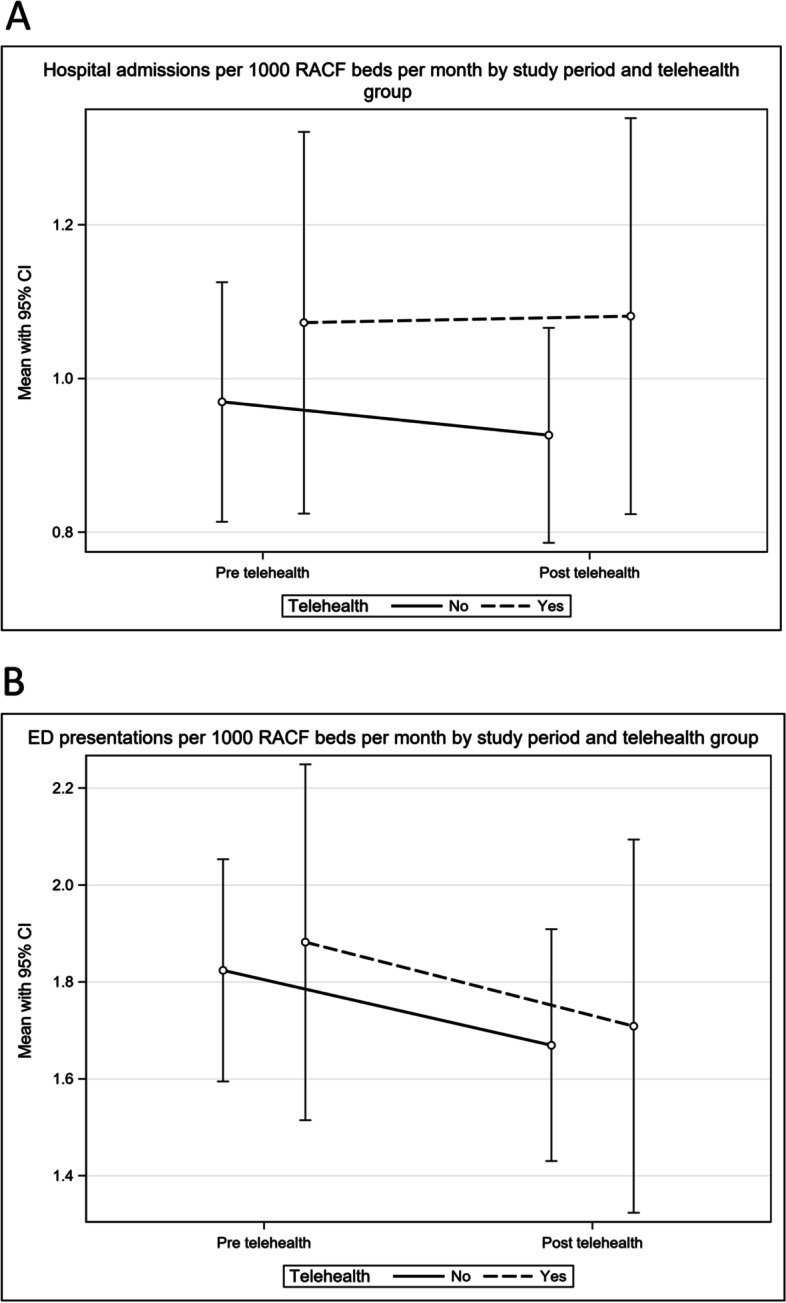
Hospital admissions (**A**) and ED visits (**B**) per 1000 RAC beds per month by study period and group

There was no significant difference in hospital admission or ED visits after the introduction of telehealth. When controlling for RAC characteristics and adjusting for clustering by RAC, RACs receiving the telehealth strategy had an approximately 2% lower admission rate during the post-telehealth phase compared to the pre-telehealth phase; RACs that did not receive the telehealth strategy had an approximately 3% higher admission rate during the post-telehealth phase compared to the pre-telehealth phase. Therefore, the admission rate was 5% lower for RACs receiving the telehealth strategy compared to those not receiving the telehealth strategy (Table 3 and Fig. 1A), however, the difference between the two groups was not statistically significant.

**Table 3:** Regression results from GLMM models for ED visits and hospital admissions

	Parameter	Univariate incident Rate Ratio (95% CI)	Adjusted Incident Rate Ratio (95% CI)	Adjusted Type III *p*-value
**ED visits**	Change from pre to post (Telehealth group)	0.89 (0.53,1.48)	0.89 (0.53,1.47)	.
	Change from pre to post (Control group)	0.94 (0.78,1.13)	0.94 (0.78,1.13)	.
	Interaction term	0.95 (0.55,1.62)	0.95 (0.55,1.62)	0.8387
**Hospital admissions**	Change from pre to post (Telehealth group)	0.98 (0.55,1.77)	0.98 (0.55,1.77)	.
	Change from pre to post (Control group)	1.03 (0.75,1.42)	1.03 (0.75,1.41)	.
	Interaction term	0.95 (0.49,1.85)	0.95 (0.49,1.85)	0.8864

ED visit rates were approximately 11% lower during the post-telehealth phase compared to the pre-telehealth phase; RACs that did not receive the telehealth strategy had an approximately 6% lower ED visit rate during the post-telehealth phase compared to the pre-telehealth phase. However, the difference between the two groups in the change in ED visit rates from the pre to post-telehealth was not statistically significant, which was approximately 5% lower for RACs receiving the telehealth strategy compared to those not receiving the telehealth strategy (Table 3 and Fig. 1B).

The mean monthly rates of ED visits and hospital admissions per 1000 beds per month, for each of the intervention conditions and by telehealth group are shown in Table 4. The rates of ED visits and hospital admissions were similar across groups. There was limited difference in the change in ED visits and admissions over time between the two telehealth groups, which supports the results from the GLMM models.

**Table 4:** Rate of ED visit and hospital admissions per 1000 RAC beds per month

		No Telehealth		Telehealth	
Variable		Pre -intervention control	Post-intervention control	Pre-intervention	Post-intervention
ED visits per 1000 RAC beds per month	mean (SD)	1.82 (1.22)	1.67 (1.28)	1.88 (1.54)	1.71 (1.62)
	median (Q1, Q3)	1.66 (0.89, 2.49)	1.33 (0.73, 2.12)	1.59 (0.71, 2.83)	1.26 (0.53, 2.55)
Hospital admissions per 1000 RAC beds per month	mean (SD)	0.97 (0.83)	0.93 (0.75)	1.07 (1.04)	1.08 (1.08)
	median (Q1, Q3)	0.68 (0.34, 1.29)	0.69 (0.35, 1.29)	0.79 (0.37, 1.54)	0.79 (0.35, 1.54)

## Discussion

Video-telehealth can support programs to reduce hospitalization, providing clinical assessment and management via live video interactions with residents, their families and staff [9, 17]. It adds visual information, compared with telephone for complex patients between hospitals and RACs [15]. By providing more comprehensive clinical communication, clinicians’ decision making and more integrated transitional care can be supported [15].

This study demonstrated a 2% reduction in admissions and an 11% reduction in ED visits in the telehealth intervention RACs, compared with a 3% increase in hospital admissions in the control sites and a 6% reduction in ED visits; however, none of these differences between the two groups from pre to post telehealth phase were statistically significant. It is important to acknowledge that this study was in addition to an established RAC hospital avoidance program which was available to both control and intervention sites. It may be that adding visual data does not change clinical decision-making regarding need for urgent transfer to ED on the background of an established and effective hospital avoidance program.

Despite the expectation that telehealth should support reduction in hospitalization of RAC residents, our findings are aligned to the limited evidence to date. Some of the reported literature is lower quality, in the form of case studies only, with no control data [18] or only historical comparisons [19–22]. Grabowski [17] undertook a cluster randomised control trial with 11 nursing homes from one for-profit chain in the USA. They also reported null findings with no significant effect on hospitalization. When they undertook a post-hoc analysis, they found that more engaged nursing homes had a significant 11% reduction in hospitalization, similar to the findings in our study [13]. Stern [23] also undertook a cluster randomised control trial in 12 long term care homes for management of pressure injuries. They found an increase in hospitalization of 20% with telehealth, though again, this was not significant, *p* = 0.52.

Although there was not a change in ED visits or subsequent hospital admissions in our study, this doesn’t mean that video-telehealth consultation is not of value. We included new or returning residents to RACs in our intervention because arrival in the RAC is reported to be a time when residents are often acutely unwell and at risk of readmission [24, 25]. This non-urgent telehealth consultation was designed to allow clinical and administrative staff in both the hospital and RAC the opportunity to develop familiarity and confidence in the telehealth process without the added burden of caring for an acutely unwell resident. It has been reported that adding video to the telephone call can allow RAC clinicians to meet the resident before they arrived, and allow family who may be geographically distant from the resident to be involved in transitional care [15]. It also reported that there can be improved integration of care with nursing home and hospital staff, developing a shared understanding of the complexity of clinical need in both settings [20].

Telehealth is an important technology innovation for clinical assessment and management of older people living in RACs. It can allow residents to be clinically supported in a familiar environment. Lyketsos [20] and Moore [24] reported that telehealth supports transitional care, clinical handover through virtual communication. Staff in our study valued telehealth handover when receiving information. Gillespie suggests that telehealth can support involvement of family and carers who otherwise may have been excluded [15]. It improves equity in access to care [4, 17, 19, 21, 26]. Salles undertook a survey of GPs who referred nursing home residents to a multi-disciplinary geriatric outpatient clinic in France [19]. Concerningly, they reported that 24% of GPs responded that the patient would not have received any additional treatment at all if the telehealth clinic was not available given the frailty and complexity of residents and risk and burden of hospitalization [22].

One of the positive outcomes in the COVID-19 pandemic in Australia and the USA [27] has been temporary billing options for primary care telehealth [28]. Improving access to primary care and reducing fragmentation of care, is a better option for residents of RACs, than a service from clinicians in the ED, who don’t know them. Interestingly, in Australia, during the pandemic, the majority of telehealth consultations in general practice has been by telephone with only 1% using video [29]. Financial incentives including funding models for telehealth are seen as an important way to facilitate access and to integrate telehealth into routine care [15, 30].

Our study followed the introduction of a multicomponent intervention [13], that had already increased the RAC staff ability to manage acutely unwell residents, avoiding ED transfer and hospital admissions where possible. Relationships and collaboration had been developed as part of the ACE community of practice [31]. It may be that the ACE program had strengthened the ability for the RACs to implement telehealth but that video-telehealth consultation was not a significant advantage over the telephone support that was already being provided, particularly in changing clinical decisions about whether or not a resident should be transferred to hospital. Through increasing confidence and collaboration of a structured hospital avoidance program, video-telehealth could be added as a discretionary tool for clinicians when they require extra visual data to determine clinical assessment and management, particularly the decision to send a resident to hospital.

### Strengths and limitations

The strengths of our study include its robust methodology: we controlled for size of the RAC, access to 24 h RNs and other factors, and had a concurrent control group. It was also a real world pragmatic trial [32, 33] using hospital administrative data, rather than self-reported hospital avoidance [34, 18].

We added telehealth in addition to a well-established hospital avoidance program. The limitations are that it involved one engaged RAC organization that was prepared to collaborate with one hospital over a 28 month period and may not be generalisable to other RACs where there will be varying levels of engagement as described by Grabowski [17]. Through previous engagement with the ACE program, the RAC organization may have already reduced the numbers of ED visits that were potentially avoidable. It is likely we would have seen a bigger impact if sites had not already had access to this program. Investigating the impact of a single intervention as part of a larger multicomponent intervention is difficult and may require different methodologies for evaluation. We have not reported other important elements for evaluation, including staff satisfaction, resident and family experience, cost analysis, and readmission and these need to be a focus for future research. The protocol for this proposed larger evaluation of telehealth has been published [35]. Future research could also include the role of telehealth in transitional care, care coordination and non-urgent transfers.

## Conclusions

Video-telehealth tended to reduce hospitalization of acutely unwell residents of RACs, however, this was not significantly different as an addition to an established and structured hospital avoidance program with nursing telephone support. Given the promotion of video-telehealth, more research is required into how to make urgent video-telehealth manageable in busy RAC, primary care, and ED environments with competing patient priorities where telephone alone may be all that is required.

## Data Availability

De-identified data is available at request to the authors.

## Abbreviations

ACE: Aged Care emergency
ED: Emergency Department
RAC: Residential Aged Care
IRR: Incident rate ratio
CI: Confidence interval
LHD: Local Health District
NSW: New South Wales
RNs: Registered nurses
GLMM: Generalized linear mixed models
